# Purchasing decisions on date palm fruits: A quantitative analysis of the *Khalas* cultivar

**DOI:** 10.1371/journal.pone.0289512

**Published:** 2023-08-03

**Authors:** Mohammed Al-Mahish, Tarifa Almulhim, Maryam Alali

**Affiliations:** 1 Department of Agribusiness & Consumer Science, College of Agriculture & Food Science, King Faisal University, Al-Ahsa, Saudi Arabia; 2 Department of Quantitative Methods, Business School, King Faisal University, Al-Ahsa, Saudi Arabia; 3 College of Agriculture & Food Science Lab, King Faisal University, Al-Ahsa, Saudi Arabia; Shandong University of Science and Technology, CHINA

## Abstract

This study examines the attributes of date palm fruits that influence consumer purchasing decisions and measures the attributes’ relative importance weights for understanding consumption patterns relative to the cultivation areas. A case study was conducted for a selected date fruit, *Khalas*, which is cultivated in Saudi Arabia and ranked first in the world in exported dates. Our empirical investigation is based on utilizing a proposed quantitative analysis that integrated the entropy weighting method and binary logit models. With this survey design, 486 questionnaires were collected. Analysis results revealed a ranking list of preferred attributes, with size, mellowness, price, and color being the most valued. However, this ranking list fluctuates when different cultivated types of *Khalas* dates are available. The results also showed that consumption patterns may change in terms of preference index and shopping location. The paper concludes with a discussion of managerial implications, limitations, and future research directions.

## 1. Introduction

Since ancient times, the palm has been known as a tree characterized by many unique qualities. It is one of the most important means of food security for desert dwellers and is known as the tree of life in desert areas. Economically, dates are one of the most prominent products of the palm tree (Phoenix dactylifera L.), and they have great nutritional value because of the vital compounds they contain [1,2]. Date palm fruits contain a single seed enclosed by fibrous and parchment-like endocarp, fleshy mesocarp, and the fruit’s skin (pericarp). Different regions produce different date fruits that vary in shape, size, and color. Accordingly, they can vary in their organoleptic, physical, and chemical characteristics [3,4]. In addition, it has become an important export commodity in a number of countries around the world, but especially in the Middle East and North Africa, with Saudi Arabia at the forefront [5]. Saudi Arabia is the third largest producer of date fruits in the global market [2,6].

In Saudi Arabia, special attention is paid to the palm and date fruits sector, as it is one of their most significant agricultural sectors, and the kingdom’s exported dates achieved an increase of 7% in value and 17% in quantity during 2020 [7]. In 2021, the total value of exported dates amounted to 1.215 billion riyals for 113 countries around the world, and Saudi Arabia ranked first globally in the export of dates by value [8]. These statistics indicate the increasing price, production, and consumption of dates domestically and globally. Several date products have attractive market value in Saudi and international markets, e.g., *Ajwa*, *Safawi*, *Sukkary*, *Khalas*, and *Sheshi* [9]. One of the popular date varieties in Saudi Arabia is *Khalas*, which is a cultivar of the palm date that has brown skin and is widely grown in Saudi Arabia and the Arab Gulf region. Furthermore, the *Khalas* cultivar ranked first in terms of the number of palm trees in Saudi Arabia, with 7,903,51 trees (25%) out of 31,234,155 total palm trees. In 2019, *Khalas* palm trees produced 422,694.8 tons of dates, and 384,394.1 tons were sold [10]. Further, there is a diversity of *Khalas* date cultivation areas in Saudi Arabia, such as Qassim, Al-Ahsa, and Riyadh. Although the *Khalas* cultivar is originally from Al-Ahsa [11], it has been also cultivated in abundance in Qassim and Kharj, and its quality differs based on where it is grown. This fact has influenced consumer purchasing decisions and created more competition in the domestic market for *Khalas* dates.

In recent years, there has been a growing focus on increasing the productivity and quality of fruit trees and consumer awareness of the demand for fresh fruits. This is characterized by external (formal) and internal (eating) quality, which gives the fruit the quality and characteristics desired by the consumer [12]. In terms of nutritional value, dates have exhibited several health benefits, as they have high amounts of phytochemicals [13]. Moreover, the external attributes of dates (e.g., size and color) may improve aspects of consumer preferences [14], and the origin of different dates may lead to different attributes (e.g., price and size), which in turn affect consumer preferences [15].

To the best of our knowledge, empirical analysis of consumer preferences for purchasing *Khalas* dates with certain attributes based on place of origin is scarce. Thus, this paper seeks to reveal how a preference for certain *Khalas* dates based on their place of origin can influence consumer purchasing decisions regarding other available *Khalas* dates. Since Al-Ahsa, Qassim, and Kharj are the three major areas that cultivate and produce dates, this research focuses on these three areas in Saudi Arabia as a case study. As a result of this research, farmers and workers in this major sector can achieve consumer satisfaction, which in turn will achieve maximum economic benefits and reduce waste. Based on these considerations, this study intended to achieve the following aims: (1) explore the impact of *Khalas* date attributes on consumer preferences for purchasing decisions is the first aim; (2) rank *Khalas* date attributes from the least to most important according to the cultivation/production areas is the second aim; and (3) create a quantitative preference index to measure the impact of preference on consumer purchasing decisions is the third aim. To address these research goals, this study applied an integrated quantitative analysis based on the entropy weighting method and binary logit models.

This research contributes to the literature by providing insights into consumer preferences that indicate potential areas of marketing and producer focus for date palm fruit markets. This is one of the first empirical applications in marketing that focuses specifically on *Khalas* date attributes concurrently with cultivation areas by conducting the proposed integrated quantitative analysis. Researchers, date producers, traders, and exporters may find this research to be a suitable framework to understand the field of date palm fruit marketing. Moreover, this study will provide a scientific framework to aid date producers and traders in improving the cultivation process and understanding the attributes that affect consumption preferences. Consequently, this can enhance the ability to compete in the market in the presence of other products that have different attributes.

## 2. Background

### 2.1 Prior research and research gap

Consumers are now being considered as the major driving force in agribusiness industry development. In recent years, a greater emphasis was being placed on studying and analyzing consumers’ behavior in purchasing decision for agri-food products. In the literature, efforts to understand consumer attitudes and the relative importance of various attributes in purchasing fruits products have been widely explored. The prior framework of consumer behavior proposes that fruits products choices are the result of considering external (formal) and internal (eating) factors [16]. Regarding date palm fruits, a stream of literature discuses that the external attributes of dates (e.g., size, color, texture, and price) may improve aspects of consumer preferences [14–16]. In addition, some authors have claimed that nutritional value characteristics (e.g. texture, and health benefits) might affect consumer purchasing decision [13,16]. A further stream of literature has investigated the consumer preferences and behavior patterns in purchasing food products that are influenced by socio-demographic characteristics of consumers. [17] showed that Norwegian consumers’ willingness to pay for African dried fruit (bananas, pineapples, and man- goes) is influenced by gender and education. [18] suggested that consumers income do play a role in shaping the demand for orange fruits in Oman. [19] found that there are differences in the purchasing and consumption behavior of Greek consumers across generations for organic food products. [20] showed that the age of Italian consumers is a significant driver of willingness to accept purchase olives leaves. [21] reveled those socio-economic characteristics of consumers, the age, education, and income have positive impacts on Willingness to Pay of dairy products in China. However, there are no studies to the best of our knowledge that considering consumer preferences for purchasing fruits products, specially *Khalas* dates, with certain attributes (color, price, fillings, mellowness, and size) integrated with based on cultivation areas is limited.

In terms of methodological approaches, consumer choices of fruit products are modelled using different models in the previous research such as contingent valuation, the experimental approach and logit models [21–24]. The most recent and suitable model was the logit models for estimating for the various preference index of fruit products for studying consumer purchasing choices [16,21,25,26]. However, the previous studies in date industry were rare to propose a weighting method for measuring the importance weights of fruits products attributes and then offering a ranked list of attributes. Thus, there is need to develop an integrated methodology for estimating and analyzing consumer choices of fruit products and at the same time measuring the importance weights of fruit products’ attributes. As far as we know, this study is the first study that propose an integrated quantitative analysis based on the entropy weighting method and binary logit models.

### 2.2 Overview of the Saudi *Khalas* date market

With regard to the quality standards for *Khalas* dates, it has been found that the Ministry of Environment, Water, and Agriculture has set standard standards for date quality to help facilitate domestic and international trade, promote high-quality production, improve the economic return of producers and exporters, and protect consumer interests. The agricultural quality standards [27] for dates are used by producers, traders, importers, and exporters.

Regarding the diversity of *Khalas* date cultivation areas in Saudi Arabia, Riyadh came in first place for the number of palm trees of the *Khalas* cultivar with 3,200,668 trees, comprising 40% of the total number of *Khalas* date palm trees in the kingdom; the eastern region came in second place with a total of 2,640,307 palm trees of the *Khalas* cultivar, or 33% of the country’s total; and the area of Qassim came in third place, as it contained 1,510,776 *Khalas* date palm trees, which is 19% of the kingdom’s total [10]. *Khalas* is also one of the most highly regarded dates in the eastern region. It is considered an excellent variety and represents 15–20% of Al-Ahsa palms, and it is believed to be native to the oasis of Al-Hofuf in the land of Al-Ahsa [11]. It is harvested and consumed unripe dates and ripped dates. The problem of harvesting small sized dates, especially in the Qatif region, is one of the factors limiting dates marketing [17]. This issue reduces consumer’s demand and eventually the economic return of farmers.

## 3. Materials and methods

### 3.1. Sample and location

The data required for this research was primary data collected through a designed survey. The required sample size was determined using the Stephen Thompson equation [28].


$$n=\frac{N\times P(1-P)}{\left[\left[N-1\times \left(d^{2}÷z^{2}\right)\right]+p(1-p)\right]}$$ (1)

Where *n* is the minimum required sample size, *N* is the population size where the *Khalas* date type *i* is cultivated, i.e. the combined population in Riyadh region, Al-Qassim, and eastern region (N = 15297768) [29], *P* is a probability value that takes a value from 0 to 1 (we set it equal to 0. 5), *d* is the margin of error (equal to 0.05), and *z* is a standardized value equal to 1.96. The results of the Thompson equation revealed that the minimum sample size should be 384 respondents. Opportunely, the number of collected observations reached a total of 486 participants. The samples were taken proportionally from date consumers in central and eastern provinces of Saudi Arabia. Enumerators were employed to distribute the survey questionnaires. Ethical approval for this study was given by the Ethical Committee at King Faisal University, and the approval reference number is KFU-REC-2022-AUG–ETHICS93. Researchers informed all participants of the reason for the research being conducted, and this was included in the introduction of the designed survey. No verbal or written consent was requested from participants because the survey does not include questions related to participants’ identity and the data were collected and analyzed anonymously.

### 3.2. Questionnaire design

In this research questionnaire, all the variables (e.g., *Khalas* date types and attributes) and items used were designed based on date palm research, general knowledge of local consumers, and informal discussions with experts. The questionnaire was composed of two sections. Section one included ten questions covering the demographic background of the respondents and other variables regarding attitudes when purchasing dates. Thus, section one considered several elements: age, gender, marital status, educational level, household size, nationality, monthly income, date shopping locations, date consumption, and preference index. Section two of the questionnaire comprised five questions asking the respondents to rank their level of purchase intention for different types of date fruits based on their area of cultivation with respect to various attributes. In this research, the three most cultivated types of *Khalas* date fruits [10] offered as alternatives in the ranking procedure were Al-Ahsa, Al-Kharj, and Al-Qassim *Khalas* dates. In addition, five date selection attributes were reflected in this study: color, price, fillings, mellowness, and size. The ranking questions were measured using five-point linguistic variables, from very high important (VH) to very low important (VLI). Table 1 shows the linguistic variable measurements and their equivalent numerical scales [30].

**Table 1:** Linguistic variable for the comprising ranking.

Linguistic term	Numerical Scale
Very High Important (VHI)	100
High Important (HI)	80
Medium Important (MI)	60
Low Important (LI)	40
Very Low Important (VLI)	20

### 3.3. Data analysis: An integrated quantitative analysis

**Fig 1:**
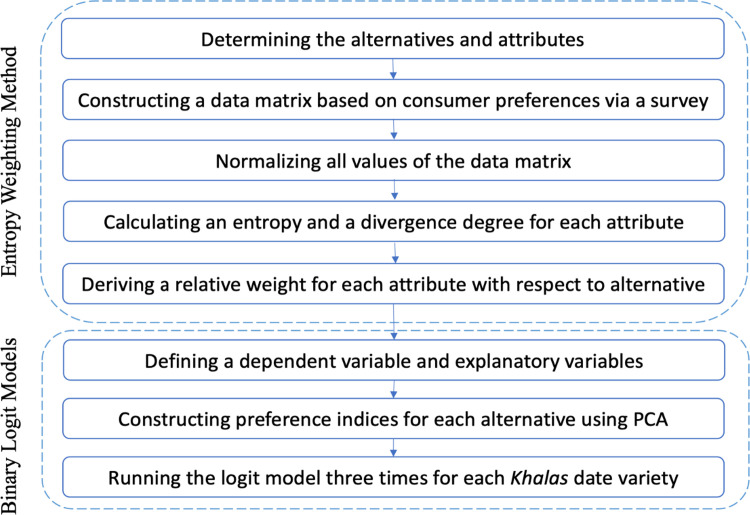
A flowchart of the integrated quantitative analysis framework.

This research consisted of an integrated quantitative analysis based on the entropy weighting method and binary logit models, which were proposed to achieve the aims of this study. The first and second aims were accomplished by applying the entropy weighting method, and binary logit models were employed to accomplish the first and third aim. Fig 1 shows a flowchart of the integrated quantitative analysis framework.

Weighting methods are commonly used for studying the preferences of online consumers by measuring the importance weights of attributes and then offering a ranked list of attributes [31]. In the literature, there are several weighting methods. SWING SMARTS, SMARTER, direct rating, Criteria Importance Through Inter-criteria Correlation, and the entropy weighting method were recent methods used in survey-based preference elicitation [32,33]. In this study, the entropy weighting method was adopted, which has been introduced to information theory to measure the amount of useful information in data provided by consumers. It focuses on the discrimination among data to determine the weighing of different attributes [34]. The calculation process for the entropy weighting method can be expressed as follows [31,33]:

With respect to *A*_*i*_, *i* = 1,2,3 denotes the number of the alternatives (here alternatives are three different kinds of *Khalas*: Al-Ahsa, Al-Kharj, and Al-Qassim dates). Answers provided by the respondents (date consumers) in the online survey were extracted from the survey database and saved in the data matrix *X*, consisting of the values *x*_*nm*_ as *T* = [*t*_*ij*_]_*M*×*N*_, *j* = 1,…*N* denotes the number of the attribute *B*_*n*_ (here *N* = 5, indicates five date selection attributes: color, price, fillings, mellowness, and size), and *i* = 1,…*M* denotes the number of the respondents. The weighting vector *W* represents the relative importance weights of *B*_*j*_ selection attributes and can be expressed as follows: *W* = {*w*_1_,*w*_2_,….*w*_*N*_}. After elicitation of consumer preferences, the decision matrix *T* will normalize as follows:

$$s_{ij}=\frac{t_{ij}}{\sum_{i=1}^{M}t_{ij}}$$ (2)

Then, an entropy *e*_*j*_ will be calculated using the following equation:

$$e_{j}=-{\left(\ln\left(\frac{1}{M}\right)\right)}^{-1}\sum_{i=1}^{M}s_{ij}\;\ln\;s_{ij}$$ (3)

Where ${\left(\ln\left(\frac{1}{M}\right)\right)}^{-1}$ is the entropy constant, which assures 0 ≤ *e*_*j*_ ≤ 1. Afterward, the degree of divergence *d*_*j*_ of the inherent information of each attribute *B*_*j*_ will be defined as:

$$d_{j}=1-e_{j}$$ (4)

Lastly, the relative importance weight *w*_*j*_ for each attribute *B*_*j*_ will be obtained using:

$$w_{j}=\frac{d_{j}}{\sum_{j=1}^{n}d_{j}}$$ (5)

In order to know how consumer preference for a certain type of *Khalas* date may influence their purchasing decisions for other types of *Khalas* dates, we will use a binary logit models specified below:

$$L_{A_{i}}=\ln\left(\frac{P_{i}}{1-P_{A_{i}}}\right)=\beta_{0}+\beta_{1}X_{1}+\cdots +\beta_{k}X_{k}$$ (6)

Where *L_A_i__* is the logit representing consumer purchasing decisions of *Khalas* type $A_{i},\ln\left(\frac{P_{A_{i}}}{1-P_{A_{i}}}\right)$ is the log of odds ratio, *β*_0_ is the intercept, *β*_1 …_ *β*_*k*_ are parameters to be estimated, and *X*_1 …_ *X*_*k*_ are vectors of independent variables. We will run the logit model three times, where the dependent variable will be consumer purchasing decisions with *Khalas* type *A*_*i*_. The explanatory variables will be the consumer preference index for type *A*_*i*_ of *Khalas* dates and other socio-economics factors that may influence consumer purchasing decisions. The preference indices will be constructed using a principal component analysis (PCA).

## 4. Results

### 4.1. General analysis

The sampling process achieved a sample size of 486 useable responses. Statistical analyses for the data collected from the sample were conducted using Microsoft Excel and R software. Table 2 illustrates the respondent profiles and summary statistics of the key variables. Descriptive statistics for the sample (Table 2) reveal a slight majority of male respondents (55%), with fewer female respondents (45%). Most respondents (63%) hold a university degree. Around 38% of them purchase *Khalas* dates from date markets, and about a quarter of them (25.10%) purchase *Khalas* dates directly from the farm owner.

**Table 2:** Summary statistics of the study’s key variables.

Variable	N (%)	Variable	Min	Mean	Max	Sd
Gender: Male Female	267 (54.94%) 219 (45.06%)	Age Household size	14 0	35.1 5.564	70 18	14.09 2.795
Marital Status: Married Unmarried	295 (60.70%) 191 (39.30%)	Monthly Income	0	12128	60000	13397
Education: Postgraduate Studies Bachelor Associate Degree High School or less	47 (9.67%) 306 (62.96%) 45 (9.26%) 88 (18.11%)	*Al-Ahsa* Preference Index *Al-Kharj* Preference Index	2.23 2.23	8.79 6.61	11.15 11.15	1.97 2.21
Date Shopping Location: Date Markets From the farm owner From their own farm Supermarket Other	185 (38.07%) 122 (25.10%) 92 (18.93%) 64 (13.17%) 23 (4.73%)	*Al-Qassim* Preference Index	2.23	7.24	11.17	2.46
*Al-Ahsa Khalas* dates Consumption: Yes No	408 (83.95%) 78 (16.05%)					
*Al-Kharj Khalas* dates Consumption: Yes No *Al-Qassim Khalas* dates Consumption: Yes No	111 (77.16%) 375 (22.84%) 186 (61.73%) 300 (38.27%)					

Also, we test the null hypothesis that the mean attributes (color, price, fillings, mellowness, and size) are equal for the three cultivation regions (Al-Ahsa, Al-Kharj, amd Alqassim). Thus, we conducted a one-way ANOVA test and Kruskal-Wallis test because the results of Shapiro-Wilk normality test (available upon request) showed that normality assumption did not hold. Nonetheless, the results of both ANOVA and Kruskal-Wallis test rejected the null hypotheses as stated in Table 3. The results show that consumers assign different level of the mean attributes (color, price, fillings, mellowness, and size) with three cultivation regions (Al-Ahsa, Al-Kharj, amd Alqassim).

**Table 3:** Decision based on ANOVA & Kruskal-Wallis test.

Null Hypothesis	Decision Based on ANOVA & Kruskal-Wallis test
Mean color score is equal for the three cultivation regions	Reject *H*_0_
Mean Price score is equal for the three cultivation regions	Reject *H*_0_
Mean Filings score is equal for the three cultivation regions	Reject *H*_0_
Mean mellowness score is equal for the three cultivation regions	Reject *H*_0_
Mean size score is equal for the three cultivation regions	Reject *H*_0_

### 4.2.Attribute importance weighting and ranking

In this research, the entropy weighting method was used to measure the relative weights for selection attributes of *Khalas* dates and then rank them. Two scenarios were examined for weighting and ranking the attributes. The first related to different cultivated types of *Khalas* dates (alternatives), since the attributes of *Khalas* dates vary from one cultivated region to another [35]. The second scenario was with respect to *Khalas* date fruits in general. Weighting and ranking of the attributes for both scenarios are illustrated in Table 4.

**Table 4:** Weighting and ranking of the attributes.

		Attributes				
With respect to		Color	Price	Fillings	Mellowness	Size
*Al-Ahsa Khalas*	Weight	0.20025	0.20016	0.19912	0.20026	0.20021
	Rank	2	4	5	1	3
*Al-Kharj Khalas*	Weight	0.20004	0.20017	0.19966	0.20002	0.20011
	Rank	3	1	5	4	2
*Al-Qassim Khalas*	Weight	0.20001	0.20017	0.1994	0.20006	0.20036
	Rank	4	2	5	3	1
For all kinds of *Khalas*	Weight	0.20009	0.20014	0.19939	0.20018	0.20020
	Rank	4	3	5	2	1

**Fig 2:**
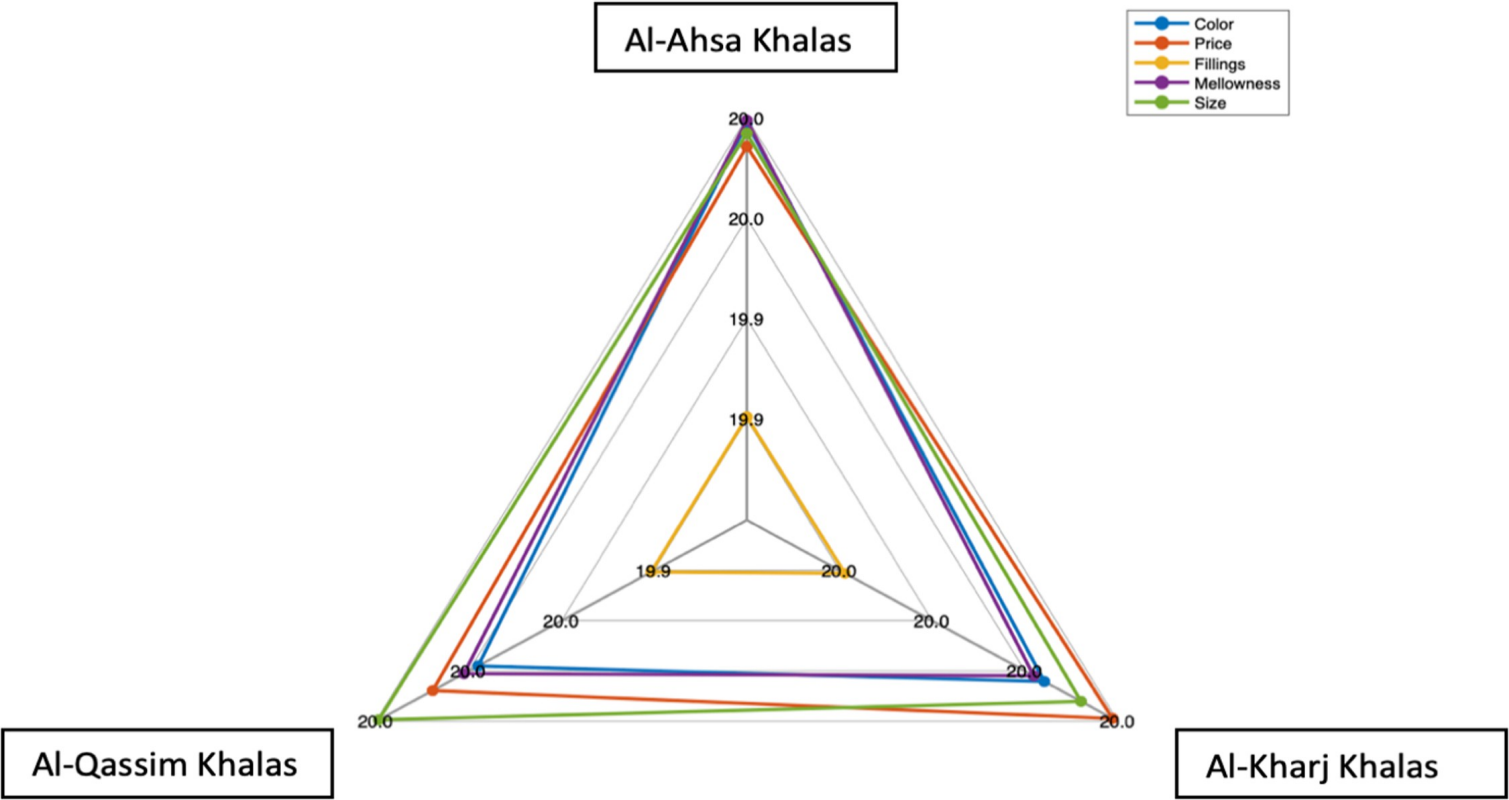
Comparison of attributes’ ranks with respect to different kinds of dates.

Regarding the first scenario, ranking *Khalas* date attributes from the least to the most important according to the cultivation areas is illustrated in Table 4, and visual comparisons are shown in Fig 2. The least important attribute is fillings, while the most important attributes are mellowness, price, and size for Al-Ahsa, Al-Kharj, and Al-Qassim *Khalas* dates, respectively. From this analysis, it is clear that the weights and ranks are inconstant in terms of four attributes: color, price, mellowness, and size. Accordingly, consumer purchasing decisions are differentiated by these four attributes when different cultivars of *Khalas* dates are available in the market, while the fillings attribute does not influence consumer purchasing decisions.

**Fig 3:**
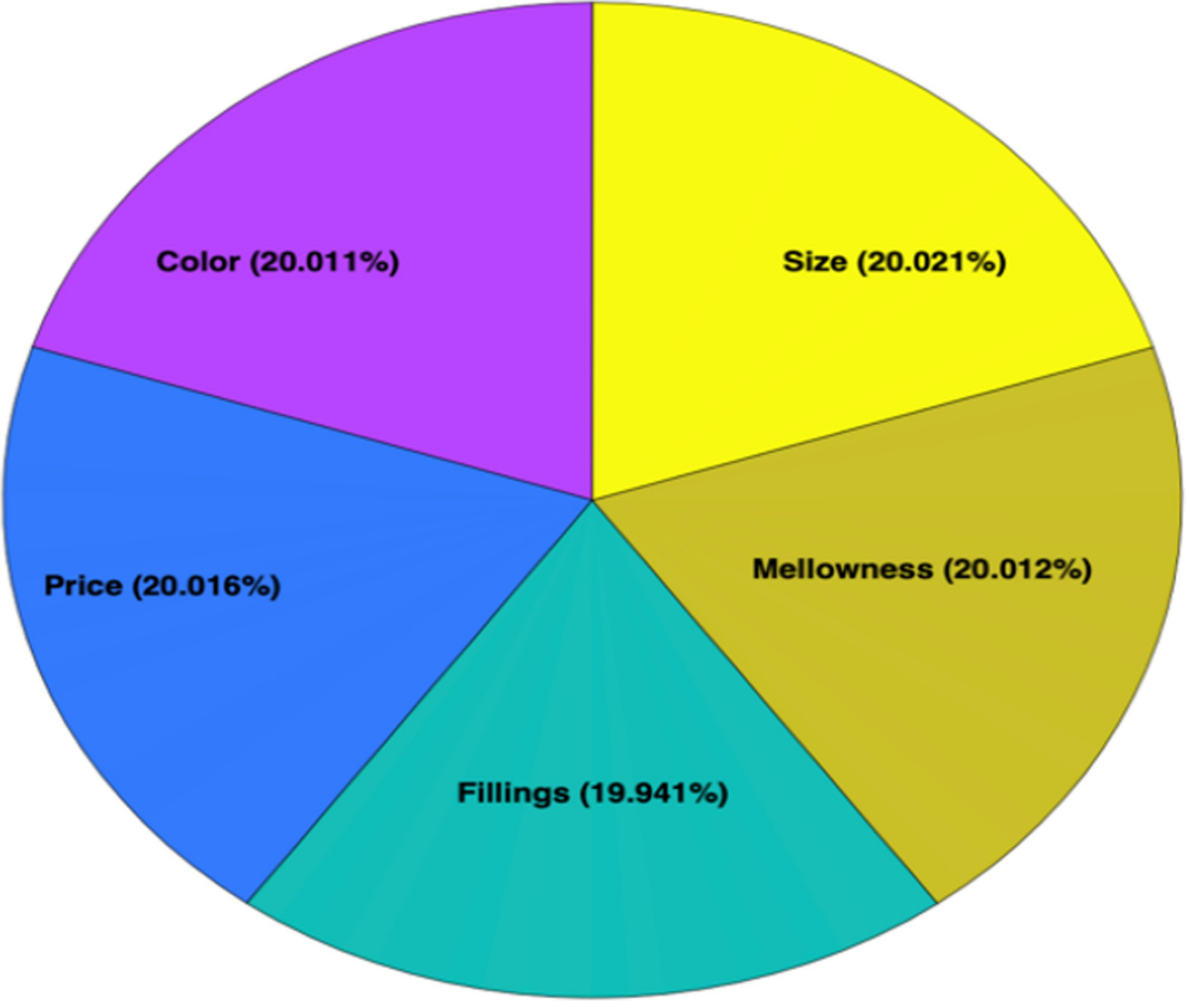
The overall results of weighting the attributes.

Regarding the second scenario, weighting and ranking of the attributes is shown in the last two rows in Table 4, and visual illustration is provided in Fig 3. The displayed results express that overall, the ranking of importance attributes that affect consumer preferences is in order of size, mellowness, price, and color. Like the first scenario’s results, the fillings are not an important attribute for consumers to consider when deciding to purchase *Khalas* dates.

### 4.3.Consumer preference index

The PCA was applied to construct a single index by utilizing *Khalas* date attributes *B*_*j*_: color, price, fillings, mellowness, and size. The results of the PCA are reported in Table 5.

**Table 5:** Principal components analysis for Khalas dates varities in Saudi Arabia.

	Component 1	Component 2	Component 3	Component 4	Component 5
Al-Ahsa Eigenvalue	3.049	0.627	0.570	0.406	0.348
Proportion of Variance	0.61	0.13	0.11	0.08	0.07
Cumulative Proportion	0.61	0.74	0.85	0.93	1
Al-Kharj Eigenvalue	2.610	0.869	0.673	0.619	0.230
Proportion of Variance	0.52	0.17	0.13	0.12	0.05
Cumulative Proportion	0.52	0.70	0.83	0.95	1
Al-Qassim Eigenvalue	2.579	0.749	0.669	0.538	0.466
Proportion of Variance	0.52	0.15	0.13	0.11	0.09
Cumulative Proportion	0.52	0.67	0.80	0.91	1

Based on the results of the PCA, we selected the first components for the three Khalas dates varities because their eigenvalues are greater than one. Thus, we construct the preference indices for the Khalas dates variaties by expressing the components as a linear combination of the observed variables as below:

Al-AhsaPreferenceIndex=0.429×Price+0.471×Color+0.395×Fillings+0.477×Mellones+0.459×Size (7)

Al-KharjPreferenceIndex=0.462×Price+0.473×Color+0.399×Fillings+0.485×Mellones+0.411×Size (8)

Al-QassimPreferenceIndex=0.463×Price+0.446×Color+0.414×Fillings+0.429×Mellones+0.482×Size (9)

Factors influencing consumer purchasing decisions for *Khalas* dates by area of cultivation were estimated with binary logit models. The results of the three models representing each area of cultivation (Al-Ahsa, Al-Kharj, and Al-Qassim) are shown in Table 6.

**Table 6:** Factors influencing consumer consumption decisions for Khalas dates.

Independent variables	Consuming Al-Ahsa *Khalas* dates	Uniform Sample Al-Ahsa	Consuming Al-Kharj *Khalas* dates	Uniform Sample Al-Kharj	Consuming Al-Qassim *Khalas* dates	Uniform Sample Al-Qassim
Intercept	-2.671** (1.081) [0.096]	-5.962*** (1.545) [0.003]	-1.774* (0.932) [0.169]	0.459 (0.155) [1.582]	-1.076 (0.803) [0.341]	0.147 (1.445) [1.159]
Al-Ahsa *Khalas* Preference	0.790*** (0.099) [2.205]	0.936*** (0.151) [2.550]	-0.331*** (0.007) [0.717]	-0.301** (0.117) [0.740]	-0.217*** (0.062) [0.805]	-0.178 (0.115) [0.837]
Al-Kharj *Khalas* Preference	-0.180* (0.094) [0.834]	-0.156 (0.128) [0.855]	0.836*** (0.113) [2.308]	0.819*** (0166) [2.268]	-0.184** (0.073) [0.832]	0.071 (0.145) [1.073]
Al-Qassim *Khalas* Preference	-0.090 (0.093) [0.913]	-0.174 (0.119) [0.841]	-0.396*** (0.091) [0.672]	-0.341** (0.141) [0.711]	0.546*** (0.007) [1.726]	0.466*** (0.136) [1.593]
Age	0.012 (0.012) [1.012]	0.039** (0.016) [1.041]	0.0118 (0.010) [1.011]	-0.018 (0.015) [0.982]	-0.023** (0.009) [0.978]	-0.065*** (0.019) [0.938]
Income	-0.000007 (0.000011) [0.999]	0.000008 (0.0001) [1.000]	0.00002** (0.00001) [1.000]	0.00003* (0.00001) [1.000]	0.000006 (0.000009) [1.000]	0.000 (0.000) [1.000]
Household Size	-0.060 (0.054) [0.941]	0.053 (0.072) [0.948]	-0.035 (0.048) [0.965]	-0.106 (0.074) [0.899]	0.094** (0.039) [1.099]	0.124* (0.071) [1.132]
Shopping Location:						
From farm owner	0.563 (0.456) [1.756]	-0.032 (0.629) [0.968]	-0.810** (0.036) [0.444]	-0.420 (0.510) [0.657]	-0.591** (0.297) [0.554]	-1.439** (0.586) [0.237]
From their own farm	0.906* (0.532) [2.475]	0.067 (0.693) [1.069]	-0.728* (0.414) [0.482]	-0.692 (0.576) [0.500]	-0.828** (0.336) [0.437]	-2.155*** (0.653) [0.116]
Supermarkets	-0381 (0.405) [0.682]	-0.123 (0.532) [0.884]	0.357 (0.354) [1.429]	1.470** (0.706) [4.348]	0.387 (0.387) [1.473]	0.701 (0.701) [2.016]
Other	0.512 (0.789) [1.669]	0.519 (1.022) [1.681]	-1.247 (0.762) [0.287]	-1.509 (0.943) [0.221]	-0.727 (0.561) [0.484]	-0.783 (0.858) [0.457]
Education:						
High school or less	-0.346 (0.428) [0.707]	0.509 (0.559) [1.052]	0.632* (0.349) [1.882]	0.654 (0.515) [1.923]	0.300 (0.290) [1.350]	-0.769 (0.578) [0.463]
Diploma	0.146 (0.584) [1.157]	0.404 (0.707) [1.499]	-0.108 (0.481) [0.89]	-0.346 (0.674) [0.708]	-1.163** (0.467) [0.313]	-1.643** (0.836) [0.193]
Graduate studies	-1.260** (0.525) [0.283]	-0.815 (0.639) [0.443]	0.271 (0.493) [1.311]	0.899 (0.868) [2.457]	0.368 (0.425) [1.446]	0.544 (0.838) [1.723]

Note: ***, **, and * indicate a significance level of 1%, 5%, and 10%, respectively. Number in parenthesis () are standard errors, and numbers in brackets [] are odds ratios.

As expected, the results show that consumer preferences for a certain type of date increases the odds of consuming that particular date, which is clearly observed through the highly significant own-preference coefficients for Al-Ahsa, Al-Kharj, and Al-Qassim *Khalas* dates, respectively. Conversely, a one-unit increase in the consumer preference score for Al-Kharj *Khalas* dates indicates a decrease in the odds of consuming Al-Ahsa *Khalas* dates by 17%. Furthermore, a one-unit increase in the consumer preference score for the Al-Ahsa and Al-Qassim dates indicates a decrease in the odds of consuming Al-Kharj *Khalas* dates by 28% and 33%, respectively. The same pattern is observed when it comes to the consumer decision to consume Al-Qassim *Khalas* dates: A one-unit increase in the consumer preference score for Al-Ahsa and Al-Kharj *Khalas* dates shows a decrease in the odds of consuming Al-Qassim *Khalas* dates by 19.5% and 17%, respectively. Overall, the results indicate that as the own-preference coefficient score for type *A*_*i*_ dates increases by one point, the odds of consuming the same type also increases. Conversely, the cross-preference coefficients indicate that a one-unit increase in the preference score for type *A*_*i*_ dates decreases the odds of consuming other types of dates.

Consumers who buy their dates directly from farm owners are less likely, on average, to consume Al-Kharj and Al-Qassim dates. Consumers who rely on their own farm as their main source of *Khalas* dates also have lower odds of consuming Al-Kharj and Al-Qassim *Khalas* dates, while they have higher odds of consuming Al-Ahsa *Khalas* dates. This can be attributed to the fact that the prices of Al-Kharj and Al-Qassim *Khalas* dates are higher than Al-Ahsa *Khalas* dates, so it is more profitable to sell Al-Kharj and Al-Qassim *Khalas* dates than Al-Ahsa *Khalas* dates. As results, the consumption decisions of consumers affected by the consumer’s preferred shopping location when they intend to purchase the *Khalas* dates.

Furthermore, as the household size increases the odds of purchasing Al-Qassim Khalas dates decreases. Conversely, older consumers and holders of associate degrees are less likely to consume Al-Qassim Khalas dates. In addition, consumers whose level of education is high school or less have higher odds of purchasing Al-Kharj Khalas dates while consumer with graduate degrees have lower odds of purchasing Al-Ahsa Khalas dates. The odds ratios in Table 6 also reveal that income does not influence the odds of consuming all the varieties of Khalas dates under investigation. This is attributed to the fact that dates are necessary commodity in Saudi Arabia and increases in income does not significantly increase dates consumption.

In order to conduct robustness check on the estimated results, we re-estimate the model with uniform samples, i.e. we created subsamples from the original data for the three dates varieties with equal number of consumers and non-consumers (111 consuming participants and 78 non-consuming participants). The results of the uniform samples in Table 6 shows that the significance level has changed for some of the preference variables. However, almost all the signs of the preference coefficients remained the same, which confirms that our results are robust.

## 5. Discussion and conclusion

This research explored the expressed preferences of a sample of 486 consumers in Saudi Arabia regarding purchasing decisions on a specific type of palm date product known as *Khalas* dates. In general, when consuming *Khalas* fruits from different areas of origin and cultivation, consumers were mainly influenced by attributes such as color, price, fillings, mellowness, and size. The proposed quantitative analysis, which integrated the entropy weighting method and binary logit models, was applied to study the purchasing decisions on *Khalas* date palm fruits.

Several of the findings are managerially and theoretically relevant. First, producers and traders should understand their targeted consumers since their preferences may change based on the varies date attributes. Second, the size, mellowness, price, and color attributes of dates, respectively, might be considered the preferred attributes of consumers. Therefore, producers that are active in the *Khalas* date industry may adapt their products to meet the preferences of their consumers. Some attributes of the product, especially sensory attributes (i.e., size, mellowness, and color), can be improved. As stated in the literature, there are new techniques to significantly improve the sensory attributes of cultivars. For instance, solar drying methods [36], automated irrigation systems [37], and the use of mycorrhizal fungi [38]. In terms of price attributes, financial and economic studies must be conducted by traders and exporters to understand the *Khalas* date market. Third, the results from the binary logistic regression models reveal that there is a direct positive relationship between consumer preference for a specific type of *Khalas* date based on its place of origin and the odds of consuming that specific type. However, this correlation decreases when there are two types of *Khalas* dates. Furthermore, consumer consumption decisions regarding *Khalas* dates is affected by the consumer’s preferred shopping location. Thus, marketing studies should be conducted frequently and include consumer preference indexes, income, shopping location, etc., to understanding purchasing decision patterns correctly. Generally, all previous results have proved the importance of running scientific marketing studies to explore the strengths that need to be supported and weaknesses that need to be addressed regarding purchasing decisions on date palm fruits. Finally, although our study focused on *Khalas* date fruits, the proposed quantitative analysis is likely to be relevant in other consumption contexts.

There are some limitations that can be improved by future research. First, the questionnaire was applied to a relatively small geographical area (consumers from central and eastern provinces of Saudi Arabia). In addition, this research only reflects five attributes of dates. Thus, researchers should incorporate more attributes and expand future research with a larger sample size. Second, the study only attempted to explore the preferences of consumers regarding purchasing decisions on date palm fruits within certain environments and did not address the uncertainty and lack of precision associated with consumer judgment and preferences. In future research, it would be interesting to utilize fuzzy set theory to study this topic with the inclusion of consumer preferences and decisions within uncertain environments. Third and most importantly, the collected consumption responses regarding Khalas dates varieties were heterogenous. We recommend for future research to consider two potential solutions in order to address potential consumption heterogeneity. The first solution is to conduct discrete choice experiment and use models that take into account consumers heterogeneity such as mixed logit model and latent class models [39]. The second recommended solution is to adjust the imbalance in consumption patterns using resampling methods such as under-sampling (decreasing majority class consumption observations to be equal to minority consumption class) or oversampling (increasing minority class consumption observations to reach to the majority class consumption) [40,41]. Finally, while these limitations hinder and make the generalizability of the findings questionable, there are promising opportunities for strengthened results with further studies.

## Supporting information

S1 File The dataset in this file was collected from respondents residing in Eastern and Central region in Saudi Arabia. (XLSX)

## References

[1] Al-Alawi R, Al-Mashiqri JH, Al-Nadabi JSM, Al-Shihi BI, Baqi Y. Date palm tree (Phoenix dactylifera L.): Natural products and therapeutic options. Frontiers in Plant Science. 2017. doi: 10.3389/fpls.2017.00845 28588600 PMC5440559

[2] Sarraf M, Jemni M, Kahramanoğlu I, Artés F, Shahkoomahally S, Namsi A, et al. Commercial techniques for preserving date palm (Phoenix dactylifera) fruit quality and safety: A review. Saudi Journal of Biological Sciences. 2021. doi: 10.1016/j.sjbs.2021.04.035 34354425 PMC8324939

[3] Barghini P, Di Gioia D, Fava F, Ruzzi M. Vanillin production using metabolically engineered Escherichia coli under non-growing conditions. Microb Cell Fact. 2007;6. doi: 10.1186/1475-2859-6-13 17437627 PMC1857700

[4] Cherif S, Le Bourvellec C, Bureau S, Benabda J. Effect of storage conditions on ‘Deglet Nour’ date palm fruit organoleptic and nutritional quality. LWT. 2021;137. doi: 10.1016/j.lwt.2020.110343

[5] Mohammed R, EL-Bateh F. Analysis of The Competitive Performance of Egyptian Dates in Global Markets in Light of An Extension Strategy to Develop Agricultural Extension Performance for The Production And Circulation of Egyptian Dates (A Case Study in The New Valley Governorate). J Agric Econ Soc Sci. 2021;12. doi: 10.21608/jaess.2021.91142.1014

[6] FAO. Faostat Crop Production Database. In: Faostat Crop Production Database. 2019.

[7] National Center for Palm and Dates. Annual Report. 2020. Available: https://ncpd.gov.sa/elnakhel/public/storage/reports/10831847831623051434_تقري2020.4.pdf.

[8] National Center for Palm and Dates. Annual Report. 2021. Available: https://ncpd.gov.sa/elnakhel/public/storage/reports/8830265571655043115_NCPD-التقريرالسنويV7.pdf.

[9] Al-Tamimi A, Alfarhan A, Rajagopal R. Antimicrobial and anti-biofilm activities of polyphenols extracted from different Saudi Arabian date cultivars against human pathogens. J Infect Public Health. 2021;14. doi: 10.1016/j.jiph.2021.10.006 34756515

[10] SGAS. Agricultural Production Survey Bulletin| General Authority for Statistics. Gas. 2019. Available: https://www.stats.gov.sa/en/1060-0.

[11] Al-bakr AJ. Palm datesits past and present and new in its cultivation,its industry and trade. Baghdad: Al-Ani Press; 1972.

[12] Albrigo LG, Stelinski LL, Timmer LW. Environmental constraints on growth, development and physiology of citrus. Citrus. 2019. doi: 10.1079/9781845938154.0078

[13] Siddiqi SA, Rahman S, Khan MM, Rafiq S, Inayat A, Khurram MS, et al. Potential of dates (Phoenix dactylifera L.) as natural antioxidant source and functional food for healthy diet. Sci Total Environ. 2020;748. doi: 10.1016/j.scitotenv.2020.141234 32798862

[14] Elsabea AMR. An economic study of processing problems for the main important varieties of dates in Saudi Arabia. Ann Agric Sci. 2012;57. doi: 10.1016/j.aoas.2012.08.009

[15] Haris A, Kamarubahrin AF, Abdul Shukor S, Kefeli Z, Ahmad ZZN, Daud SNM, et al. Is the Superfruit Dates (Phoenix Dactylifera L.) Meant for High End Consumers? Int J Fruit Sci. 2021;21. doi: 10.1080/15538362.2021.1936344

[16] El Hadad-Gauthier F, Monhoussou BB, Hammoudi A, Perito MA. European Consumers Attitudes toward Ethnic Foods: Case of Date Fruits. Foods. 2022;11. doi: 10.3390/foods11152192 35892777 PMC9331604

[17] Alphonce R, Temu A, Almli VL. European consumer preference for African dried fruits. Br Food J. 2015;117. doi: 10.1108/BFJ-10-2014-0342

[18] Harahap LM, Amanah D, Harahap DA, Jubaidah S. Factors Affecting Consumer Demand on Orange Fruit In Pantai Buaya, Langkat, Indonesia. Int J Econ Bus Manag Res. 2019;3.

[19] Kamenidou I, Stavrianea A, Bara EZ. Generational differences toward organic food behavior: Insights from five generational cohorts. Sustain. 2020;12. doi: 10.3390/su12062299

[20] Perito MA, Coderoni S, Russo C. Consumer attitudes towards local and organic food with upcycled ingredients: An Italian case study for olive leaves. Foods. 2020;9. doi: 10.3390/foods9091325 32962245 PMC7554815

[21] Zhou H, Nanseki T. Traceability System of Dairy Products and Its Impacts on Consumer Behavior in China: An Application of Multinominal Logit Model. Agricultural Innovation in Asia: Efficiency, Welfare, and Technology. Springer; 2023. pp. 149–157.

[22] Moser R, Raffaelli R, Thilmany-McFadden D. Consumer preferences for fruit and vegetables with credence-based attributes: A review. Int Food Agribus Manag Rev. 2011;14.

[23] Costa CA da, Santos JL. Estimating the demand curve for sustainable use of pesticides from contingent-valuation data. Ecol Econ. 2016;127. doi: 10.1016/j.ecolecon.2016.04.019

[24] Rihn A, Wei X, Khachatryan H. Text vs. logo: Does eco-label format influence consumers’ visual attention and willingness-to-pay for fruit plants? An experimental auction approach. J Behav Exp Econ. 2019;82. doi: 10.1016/j.socec.2019.101452

[25] Briz T, Ward RW. Consumer awareness of organic products in Spain: An application of multinominal logit models. Food Policy. 2009;34. doi: 10.1016/j.foodpol.2008.11.004

[26] Scarpato D, Rotondo G, Simeone M, Gómez A, Gutiérrez P. How can food companies attract the consumer concerned about food safety? A logit model analysis in Spain. Br Food J. 2017;119. doi: 10.1108/BFJ-12-2016-0616

[27] Ministry of Environment W and A. Agricultural Book. 2020. Available: https://www.mewa.gov.sa/ar/InformationCenter/Researchs/Reports/GeneralReports/الكتابالإحصائي2020.pdf.

[28] Thompson SK. Sample Size. Sampling. 2012. pp. 53–56. doi: 10.1002/9781118162934.ch4

[29] Saudi General Authority for Statistics. General Authority for Statistics. In: Stats.Gov.Sa/En/ [Internet]. 2019 pp. 2018–2020. Available: https://database.stats.gov.sa/home/indicator/410.

[30] Kahraman C, Keshavarz Ghorabaee M, Zavadskas EK, Cevik Onar S, Yazdani M, Oztaysi B. Intuitionistic fuzzy EDAS method: an application to solid waste disposal site selection. J Environ Eng Landsc Manag. 2017;25. doi: 10.3846/16486897.2017.1281139

[31] Lescauskiene I, Bausys R, Zavadskas EK, Juodagalviene B. Vasma weighting: Survey-based criteria weighting methodology that combines entropy and waspas-svns to reflect the psychometric features of the vas scales. Symmetry (Basel). 2020;12. doi: 10.3390/sym12101641

[32] Aubert AH, Esculier F, Lienert J. Recommendations for online elicitation of swing weights from citizens in environmental decision-making. Oper Res Perspect. 2020;7. doi: 10.1016/j.orp.2020.100156

[33] Li Q, Hu H, Ma L, Wang Z, Arıcı M, Li D, et al. Evaluation of energy-saving retrofits for sunspace of rural residential buildings based on orthogonal experiment and entropy weight method. Energy Sustain Dev. 2022;70: 569–580. 10.1016/j.esd.2022.09.007.

[34] Das S, Dutta B, Guha D. Weight computation of criteria in a decision-making problem by knowledge measure with intuitionistic fuzzy set and interval-valued intuitionistic fuzzy set. Soft Comput. 2016;20. doi: 10.1007/s00500-015-1813-3

[35] Aljobair MO, Albaridi NA, Alkuraieef AN, AlKehayez NM. Physicochemical properties, nutritional value, and sensory attributes of a nectar developed using date palm puree and spirulina. Int J Food Prop. 2021;24. doi: 10.1080/10942912.2021.1938604

[36] T. S, Al-Ismaili AM, Janitha Jeewantha LH, Al-Habsi NA. Effect of solar drying methods on color kinetics and texture of dates. Food Bioprod Process. 2019;116. doi: 10.1016/j.fbp.2019.03.012

[37] Alnaim MA, Mohamed MS, Mohammed M, Munir M. Effects of Automated Irrigation Systems and Water Regimes on Soil Properties, Water Productivity, Yield and Fruit Quality of Date Palm. Agric. 2022;12. doi: 10.3390/agriculture12030343

[38] Meddich A, Jaiti F, Bourzik W, Asli A El, Hafidi M. Use of mycorrhizal fungi as a strategy for improving the drought tolerance in date palm (Phoenix dactylifera). Sci Hortic (Amsterdam). 2015;192. doi: 10.1016/j.scienta.2015.06.024

[39] Zha D, Yang G, Wang W, Wang Q, Zhou D. Appliance energy labels and consumer heterogeneity: A latent class approach based on a discrete choice experiment in China. Energy Econ. 2020;90. doi: 10.1016/j.eneco.2020.104839

[40] Lakshmanan V, Robinson S, Munn M. Machine Learning Design Patterns. O’Reilly Media, Inc; 2020.

[41] Huyen C. Designing Machine Learning Systems. O’Reilly Media, Inc; 2022.

